# An Evaluation of the Potential Radiosensitization Effect of Spherical Gold Nanoparticles to Induce Cellular Damage Using Different Radiation Qualities

**DOI:** 10.3390/molecules30051038

**Published:** 2025-02-24

**Authors:** Monique Engelbrecht-Roberts, Xanthene Miles, Charlot Vandevoorde, Maryna de Kock

**Affiliations:** 1 Department of Medical Bioscience, Faculty of Natural Sciences, University of the Western Cape, Cape Town 7535, South Africa; 2 Radiation Biophysics Division, Separated Sector Cyclotron Laboratory, iThemba LABS (NRF), Cape Town 7100, South Africa; 3 Space Radiation Biology, GSI Helmholtzzentrum für Schwerionenforschung GmbH, Planckstraße 1, 64291 Darmstadt, Germany

**Keywords:** gold nanoparticles (AuNPs), dose enhancement effect, radiosensitizer interaction indices, linear energy transfer (LET), radiation therapy, nanomedicine

## Abstract

Global disparities in cancer prevention, detection, and treatment demand a unified international effort to reduce the disease’s burden and improve outcomes. Despite advances in chemotherapy and radiotherapy, many tumors remain resistant to these treatments. Gold nanoparticles (AuNPs) have shown promise as radiosensitizers, enhancing the effectiveness of low-energy X-rays by emitting Auger electrons that cause localized cellular damage. In this study, spherical AuNPs of 5 nm and 10 nm were characterized and tested on various cell lines, including malignant breast cells (MCF-7), non-malignant cells (CHO-K1 and MCF-10A), and human lymphocytes. Cells were treated with AuNPs and irradiated with attenuated 6 megavoltage (MV) X-rays or p(66)/Be neutron radiation to assess DNA double-strand break (DSB) damage, cell viability, and cell cycle progression. The combination of AuNPs and neutron radiation induced higher levels of γ-H2AX foci and micronucleus formation compared to treatments with AuNPs or X-ray radiation alone. AuNPs alone reduced cellular kinetics and increased the accumulation of cells in the G2/M phase, suggesting a block of cell cycle progression. For cell proliferation, significant effects were only observed at the concentration of 50 μg/mL of AuNPs, while lower concentrations had no inhibitory effect. Further research is needed to quantify internalized AuNPs and correlate their concentration with the observed cellular effects to unravel the biological mechanisms of their radioenhancement.

## 1. Introduction

A combination of therapies, including surgery, chemotherapy, radiation, and targeted and hormone therapy, is currently available to treat cancer. Despite these advances in the treatment, South Africa is ranked 50th on the World Cancer Research Fund’s list of countries with the highest cancer prevalence rates [1], and an increase of 78% is predicted by 2030 [2]. Radiation dosage is limited by the tolerance level of the healthy organs [3]; therefore, sensitizing the malignant cells by specific radiosensitizers has long been a goal of cancer researchers. The development of gold nanoparticles (AuNPs) with biocompatible characteristics has stimulated research to pursue the application of AuNPs in combination with radiation therapy [4,5,6]. AuNPs have been indicated as excellent radiosensitizers by enhancing the vulnerability of the tumor tissue to radiation exposure without damaging healthy surrounding soft tissue [4,7,8].

The most prominent results with AuNPs as radiosensitizers have been obtained with kilovoltage (kV) X-rays, which unfortunately have a limited tissue penetration depth in comparison to megavoltage (MV) X-rays. Kilovoltage X-rays are low-energy radiation sources (e. g., 150–200 kV); thus, at these energies, the highest radiation dose is deposited at the skin surface, and 90% of the dose will occur ~2 cm deep in the tissue [9]. Numerous research studies with kV X-rays have shown significant dose-enhancement effects, with increasing treatment efficacies of up to 200% via their absorption of X-ray emissions [8,10,11,12,13,14,15]. The number of studies using different radiation qualities, such as MV X-rays, protons, and even neutrons [16], is steadily growing in recent years, showing often mixed and variable results [10,17,18,19,20,21,22,23,24,25]. The rationale for the clinical use of neutron radiation beams is primarily motivated by neutrons that interact differently with matter compared to other radiation types, like X-rays or gamma rays. They cause direct nuclear reactions, leading to a different spectrum of secondary particles and energy deposition patterns in cells. This could potentially create more complex and localized damage when interacting with AuNPs, which may improve therapeutic outcomes. However, AuNPs have been indicated as excellent radiosensitizers by enhancing the vulnerability of the tumor tissue to radiation exposure without damaging healthy surrounding soft tissue [22].

AuNPs with a diameter of 100 nm or less are commonly used in radiation research applications, due to the high atomic number (Z) of gold (Z = 79), which results in considerable differences in mass energy absorption properties in contrast to soft tissue [26,27,28]. AuNPs can accumulate in tumor tissues via the enhanced permeability and retention (EPR) effect [29,30], also known as passive targeting [31]. These properties not only facilitate tumor accumulation but also enhance the potential for therapeutic effects when AuNPs are irradiated.

The dose-enhancement effect of AuNPs is based on the greater probability for photo-interactions by the photoelectric effect when irradiated by kV X-rays [32,33]. When X-rays interact with the electron clouds of atoms, particularly the inner-shell electrons, they can induce photoelectric absorption. In the case of AuNPs, this interaction ejects inner-shell electrons, creating vacancies. Berbeco and co-workers stated that AuNPs are relevant for in vitro radiotherapy research, due to the high K-edge of Au (~81 keV), which can result in the emission of short-range photoelectrons upon radiation with low liner energy transfer (LET) photons [34]. Unlike X-rays, neutrons interact with atomic nuclei instead of electrons. During fast neutron therapy, neutrons interact with the atomic nuclei of the gold, whereas X-rays interact with the orbital electrons. While X-rays lead to the direct emission of Auger electrons via photoelectric absorption, fast neutrons interact with hydrogen nuclei in biological tissues, producing recoil protons. These protons ionize surrounding tissues, creating secondary radiation effects. Neutron interaction with AuNPs can also induce nuclear reactions, resulting in indirect Auger electron production, but this process is more complex and less efficient compared to X-rays. Auger electrons have a short range, but produce densely localized ionizations and excitations within DNA, inducing poorly repairable damage (double stranded DNA breaks). Auger electrons emit a maximum energy of 0.5–25 keV and travel short distances, typically 0.02–10 µm [35]. The biological effects of Auger emitters are highly dependent upon their cellular and subcellular distribution [36]. Therefore, they are effective when the AuNPs are internalized in the cell, preferably close to the nucleus [37]. Fast neutrons do not interact with atomic electrons like photons do, but instead, the uncharged fast neutrons efficiently interact with hydrogen nuclei, producing recoil protons that ionize [38]. Therefore, the probability of photoelectric interactions increases for lower-energy photons and materials with higher atomic numbers [39,40]. This suggest that AuNPs enhance the radiotoxicity of neutron radiation more significantly than X-ray radiation [16]. In summary, AuNPs exposed to X-rays will produce Auger electrons more directly and efficiently through photoelectric absorption and subsequent electron transitions. In contrast, neutron exposure may lead to Auger electron production indirectly through nuclear reactions and radioactive decay, which is generally less efficient and involves more complex processes.

The biological effects of Auger emitters are highly dependent upon their cellular and subcellular distribution [36]. Therefore, they are effective when the AuNPs are internalized in the cell, preferably close to the nucleus [37]. Recently, AuNPs have been incorporated into boron neutron capture therapy for the assessment of their potential as drug candidates [41,42]. Although this is less efficient than the direct photoelectric effect seen with X-rays, it may still enhance the overall radiotoxicity, especially when the AuNPs are located near critical cellular structures like the nucleus. However, the exact mechanisms through which AuNPs may enhance radiotoxicity in neutron-based therapies are not yet fully understood. Beyond the classical photoelectric effect, emerging studies suggest that AuNPs also act as potent radiosensitizers through mechanisms unrelated to direct electron emission. AuNPs can catalyze reactive oxygen species (ROS) production upon radiation exposure, leading to oxidative stress and cellular damage. The presence of AuNPs has been shown to enhance radiation-induced ROS levels, further amplifying DNA and membrane damage. Studies indicate that AuNPs may disrupt the balance between ROS production and antioxidant defense mechanisms, making cancer cells more susceptible to radiation-induced oxidative damage [43,44,45,46,47,48]. AuNPs can interfere with DNA repair pathways, making tumor cells more susceptible to radiation-induced damage. They can impair key repair mechanisms such as non-homologous end joining (NHEJ) and homologous recombination (HR) by disrupting protein interactions involved in DNA damage response. This results in increased DSBs, chromosomal aberrations, and apoptotic cell death, ultimately improving the efficacy of radiation therapy. Studies have shown that by hindering repair processes, AuNPs contribute to prolonged DNA damage, increasing tumor cell sensitivity to radiation exposure [45,49,50]. When cells are treated with AuNPs, flow cytometry studies reveal that AuNPs cause cell cycle arrest in the G2/M phase, where cells are highly radiosensitive due to inefficient DNA repair. The G2/M phase is highly sensitive to radiation because the cells have a reduced ability to repair DNA damage before progressing to mitosis. By extending this phase, AuNPs enhance radiation sensitivity, making cells more vulnerable to radiation-induced damage and increasing tumor cell death [22,51]. In summary, AuNPs enhance radiation effects not only through the photoelectric effect and Auger electron emission but also by increasing oxidative stress and impairing DNA repair. X-ray-induced Auger electron production is more efficient; neutron interactions with AuNPs may still contribute to overall radiosensitization through complex secondary effects. Further studies, including simulation-based research, are needed to clarify these interactions and optimize AuNP applications across different radiation modalities.

The purpose of our study was to assess the radiosensitization potential [22] of AuNPs (5 nm and 10 nm) in various cell types for attenuated 6 MV X-rays and neutrons, in order to simulate low-energy X-rays using an attenuated 6 MV X-ray LINAC [34]. Induction of DNA double-strand breaks, cell cycle arrest, and variation in cell viability were assessed to deduce the impact of AuNPs and X-rays or neutrons on cellular integrity.

## 2. Results

### 2.1. The Characterization of 5 nm and 10 nm AuNPs

**Figure 1:**
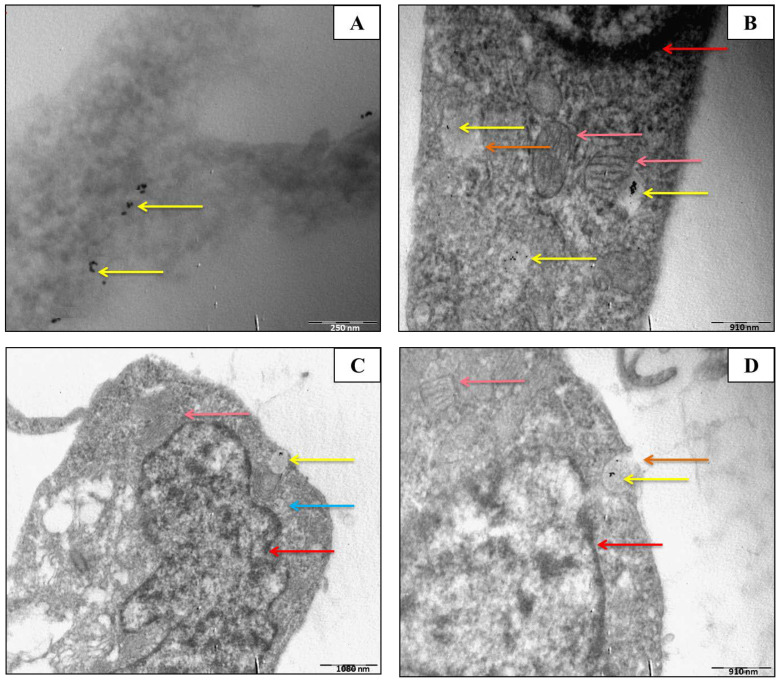
TEM micrographs of 50 μg/mL of 10 nm AuNPs within MCF-10A cells. After the 4 h incubation period, some AuNPs were observed in the vicinity of the nucleus. (**A**). AuNPs were observed in the cytoplams of the cells and near the nuclear membranes of the cells. (**B**) The AuNPs (yellow arrows) are distributed within the cytoplasm, near the nuclear membrane (red arrows). Swollen mitochondria (pink arrows) are indicative of potential AuNP-induced cytotoxic stress. The AuNPs are taken up by endocytosis, which is clearly indicated by the orange arrows. (**C**) AuNPs (yellow arrows) are located near the nuclear membrane (red arrow), with additional evidence of swollen mitochondria (pink arrow) and potential autophagosomes or vesicles (blue arrows). (**D**) AuNP uptake via endocytosis (orange arrows) is evident, with nanoparticles accumulating near the nuclear membrane (red arrow).

**Figure 2:**
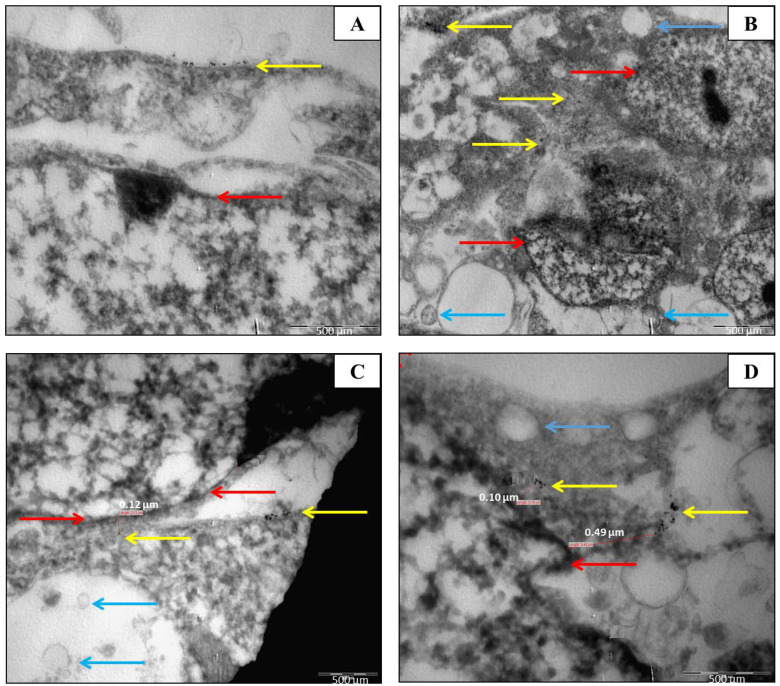
TEM micrographs of 50 μg/mL of 10 nm AuNPs within MCF-7 cells. (**A**) AuNPs (yellow arrows) are located in the cytoplasm near the nuclear membrane (red arrow). (**B**) Multiple vesicles containing AuNPs (yellow arrows) are evident, along with a large number of possible lysosomal bodies (blue arrows) observed in the MCF-7 cells. (**C**) After the 4 h incubation period, some AuNPs were observed in the vicinity of the nucleus. The red arrows indicate the nuclear membrane of the cell. The AuNPs are taken up in the cells and is seen with distances to the nuclear membrane (red arrows) measured at 0.12 μm. Possible lysosomal bodies are also visible (blue arrows). (**D**) AuNP accumulation near the nuclear membrane (red arrow) is highlighted, with distances of 0.10 μm and 0.49 μm. Lysosomal bodies (blue arrow) are also present.

The analysis of surface plasmon resonance (SPR) using UV-vis spectrometry confirmed the presence of AuNPs. The UV-vis spectra of both types of AuNPs were obtained using the Agilent 8453. Absorbance profiles were measured between 525–580 nm. Z-potential is an indication of repulsive and attractive forces between nanoparticles and can predict the long-term stability of nanoparticles in the solution. The negative Z-potential charge of the AuNPs (from −24.5 mV for 5 nm AuNPs to −23.2 mV for 10 nm AuNPs) represents the necessary repulsive forces for the particles to remain stable in solution. The Z-potentials of 5 and 10 nm AuNPs were −24.5 mV and −23.2 mV, respectively. The negative Z-potential values present the necessary repulsive forces for the particles to remain stable in the solution. Nanoparticles with Z-potential < −30 mV are regarded as strongly anionic, whereas nanoparticles with a Z-potential > +30 mV are regarded as strongly cationic. Data were obtained in phase analysis light scattering mode at 25 °C, and pH 7.4 PDI values represent the size distribution width of nanoparticles. A PDI value of 0.1–0.25 suggests that the nanoparticles have a uniform size distribution, whilst a PDI > 0.5 indicates a very broad distribution. Results showed that both sizes of AuNPs have a uniformity size width distribution. These larger sizes in diameter may be due to the agglomeration state of nanoparticles as a function of time or suspending solution. Furthermore, DLS measurements were used to determine the hydrodynamic size of citrate-coated AuNPs. DLS measurements (Z-average) show that the 5 nm AuNPs are 38.12 nm in diameter, whilst 10 nm AuNPs are 48.50 nm in diameter. These larger diameters may be due to the agglomeration state of nanoparticles as a function of time or suspending solution. TEM was used to determine the uptake and location of the AuNPs in two cell lines, namely, MCF-7 and MCF-10A cells (Figure 1 and Figure 2). In **MCF-7 cells**, AuNPs were primarily localized within membrane-bound vesicles, such as endosomes and lysosomes, indicating active endocytotic pathways. Some nanoparticles were also observed near the nuclear membrane, though none were detected inside the nucleus. Additionally, AuNPs were found scattered within the cytoplasm, suggesting partial vesicle escape.

In **MCF-10A cells**, the AuNPs exhibited similar localization patterns, with the majority confined within endosomes and lysosomes. However, fewer particles were observed near the nuclear membrane compared to MCF-7 cells. No nuclear localization was detected in either cell line, indicating that AuNPs remained within cytoplasmic compartments.

The distinct distribution patterns suggest differential uptake efficiencies between the two cell lines, potentially influencing the radiobiological response.

### 2.2. The Preliminary γ-H2AX Foci Study of PBMCs

**Figure 3:**
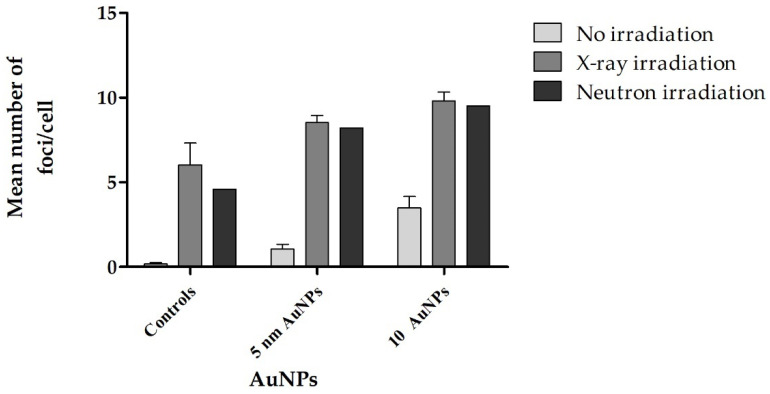
This figure shows the mean number of γ-H2AX foci per lymphocyte at 4 h post-irradiation. The plot represents the quantification of effects of isolated healthy adult lymphocytes (*n* = 3) incubated with culture media containing 50 μg/mL of AuNPs for 4 h followed by 1 Gy X-rays and 1 Gy p(66)/Be neutron radiation, respectively. Error bars represent standard error of the mean (SEM) of the different donors.

The number of foci per cell increased for cells treated with AuNPs and X-ray and p(66)/Be neutron irradiations, as compared to the controls in Fce Combined treatment (AuNPs and ionizing radiation) obtained higher endogenous foci in lymphocytes, in comparison to lymphocytes that were only treated with AuNPs alone. Significant increases (*p* < 0.05 (*) in foci were observed in the neutron-irradiated samples compared to the control.

### 2.3. The Cell Viability Assay

The MTT assay was used to assess the overall toxicity of the AuNPs in the CHO-K1, MCF-7, and MCF-10A cells. Cells were treated with different concentrations of the two types of AuNPs (2.5, 5, 10, and 50 μg/mL) for different times (4 h non-irradiated, 4 h irradiated with 4 Gy attenuated 6 MV X-rays, and 24 h non-irradiated) in triplicate

**Figure 4:**
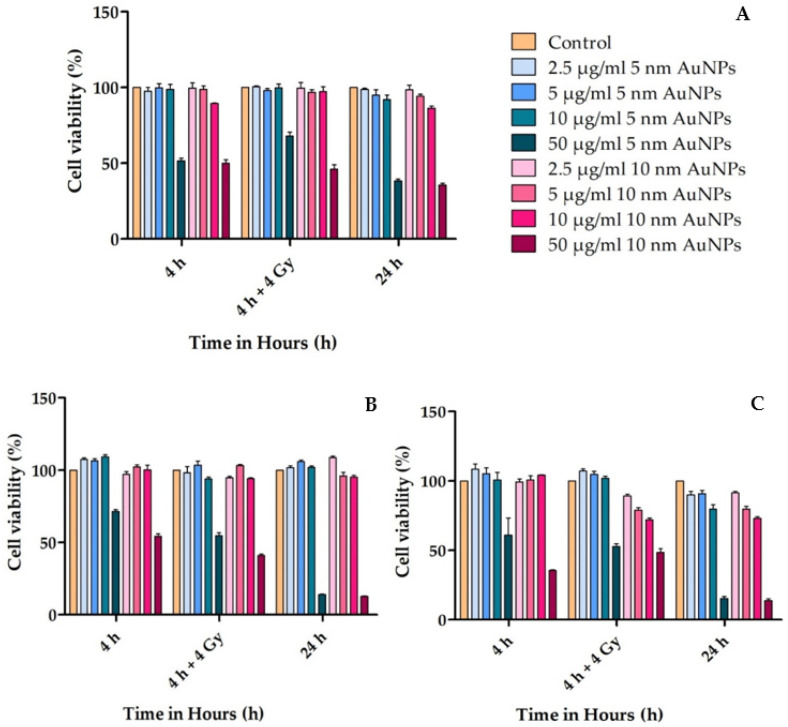
Cell viability of (**A**) CHO-K1 cells, (**B**) MCF-10A cells, and (**C**) MCF-7 cells. The figures show the percentage (%) of cell viability in four different cell lines as determined by MTT in 96-well plates: cells incubated with AuNPs for 4 h, cells incubated with AuNPs for 4 h followed by 4 Gy attenuated X-ray radiation, and cells incubated with AuNPs for 24 h. After the three time–exposure conditions (4 h, 4 h and 4 Gy, and 24 h) at 50 μg/mL, both 5 and 10 nm AuNPs caused a significant decrease in cell proliferation (*p* < 0.05).

The conclusions drawn from Figure 4 are limited by the use of a single irradiation dose; while survival curves and linear-quadratic model parameters would provide deeper insights, this study was not designed to include additional doses, focusing instead on evaluating the interaction between AuNPs and radiation at a relevant dose. Future research should explore dose-dependent effects using survival curves to better characterize the radiobiological response and underlying mechanisms.

**Figure 5:**
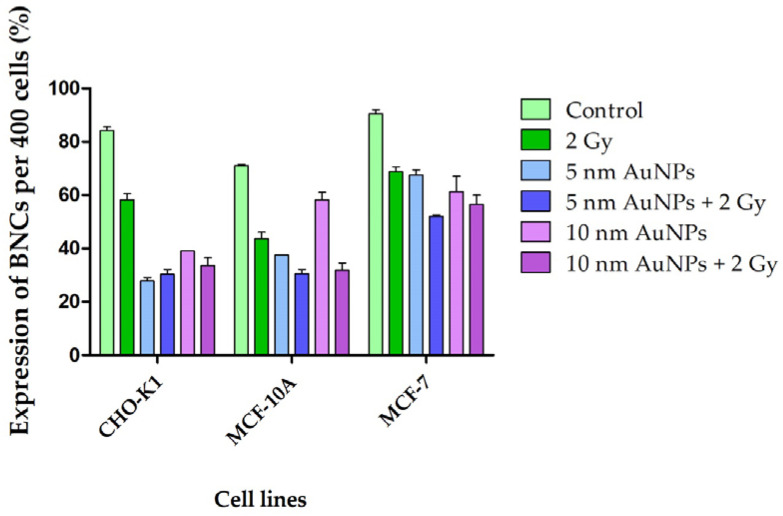
Cellular kinetics of CHO-K1, MCF-10A, and MCF-7 cell lines. Cellular kinetics of the cells was determined by scoring 400 AO-stained BNCs and expressed as a percentage (%). All cell lines treated with 50 μg/mL AuNPs and/or irradiated with 2 Gy X-rays showed an overall decrease in cellular kinetics in comparison to the control.

**Figure 6:**
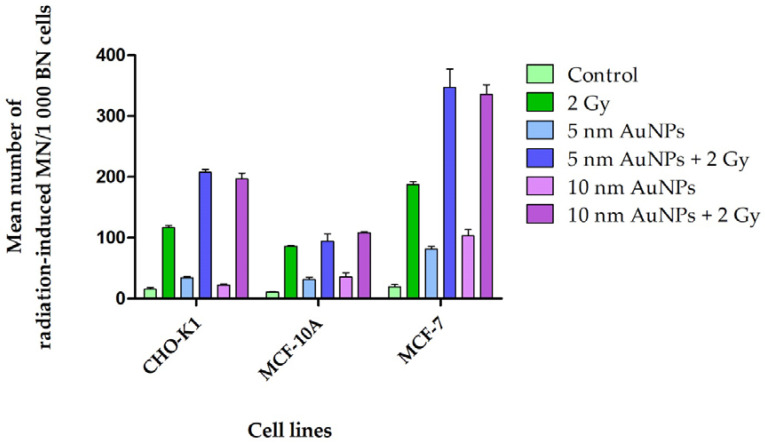
Mean number of radiation-induced MN of CHO-K1, MCF-10A, and MCF-7 cell lines. Mean cumulative frequency of MN present was determined via the CBMN assay in the cells after 4 h incubation with 50 μg/mL AuNPs followed by 2 Gy attenuated 6 MV X-ray radiation. Control cells treated with AuNPs showed a negligible number of MN, whilst a noticeable increase of MN within cells treated with AuNPs and radiated with attenuated 6 MV 2 Gy X-rays was apparent. The mean frequency of MN present determined via the CBMN assay in CHO-K1 cells after 4 h incubation with 50 μg/mL AuNPs followed by 2 Gy X-ray radiation. Control cells treated with AuNPs showed a small number of MN, whilst an increase of MN within cells treated with AuNPs and radiated with 2 Gy X-rays was apparent. The interaction indices for AuNPs and attenuated 6 MV X-rays in CHO-K1 cells are 1.6 to 1.7, thus >Unity (Unity = 1). The MCF-10A control cells treated with AuNPs showed an insignificant number of MN in control, whilst a noticeable increase of MN within cells treated with AuNPs and radiated with 2 Gy attenuated 6 MV X-rays was apparent. The interaction indices for AuNPs and 6 MV X-rays of 0.87 to 0.97 were determined for MCF-10A cells, which are <Unity (Unity = 1). The MCF-7 control cells treated with AuNPs displayed an insignificant number of MN, whilst an increase of MN within cells treated with AuNPs and radiated with 2 Gy attenuated 6 MV X-rays was apparent. The interaction indices for AuNPs and 6 MV X-rays of 1.3 to 1.4 were determined for MCF-7 cells, which are >Unity (Unity = 1).

An interaction between the AuNPs and attenuated 6 MV 2 Gy X-rays was only noted in two cell lines, namely, CHO-K1 and MCF-7. Therefore, only CHO-K1 and MCF-7 cells were used for further CBMN assay.

**Figure 7:**
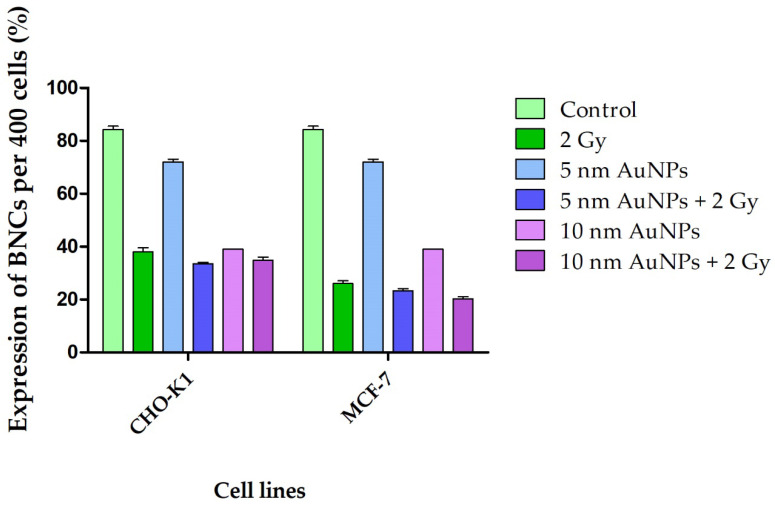
Cellular kinetics of both CHO-K1 and MCF-7 cells were determined by scoring 400 AO-stained BNCs and expressed as a percentage (%). Cells treated with 50 μg/mL AuNPs and/or irradiated with 1 Gy p(66)/Be neutrons showed an overall decrease in cellular kinetics in comparison to the control.

**Figure 8:**
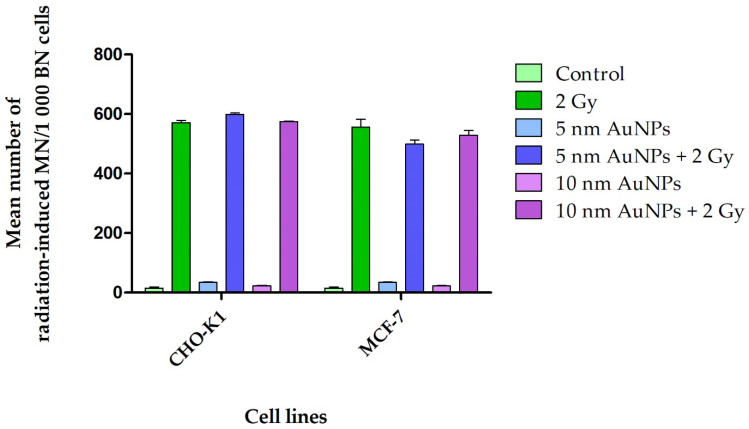
Mean number of radiation-induced MN of CHO-K1 and MCF-7 cell lines. The mean cumulative frequency of MN present were determined via the CBMN assay in CHO-K1 cells after 4 h incubation with 50 μg/mL AuNPs followed by 1 Gy p(66)/Be neutrons. Control cells treated with AuNPs displayed an insignificant number of MN, whilst a visible increase of MN within cells treated with AuNPs and radiated with 1 Gy p(66)/Be neutrons was evident. The interaction indices for AuNPs and 1 Gy p(66)/Be neutrons of 1.06 to 1.16 are lower than the interaction indices after 2 X-rays. The mean frequency of MN present was determined via the CBMN assay in MCF-7 cells after 4 h incubation with 50 μg/mL AuNPs followed by 1 Gy p(66)/Be neutrons. No difference in the number of MN within cells treated with AuNPs and radiated with 1 Gy p(66)/Be neutrons and the radiated control was noticeable. The interaction indices for AuNPs and 1 Gy p(66)/Be neutrons of 0.88 to 0.95 is lower than the interaction indices after attenuated 6 MV 2 Gy X-rays.

## 3. Discussion

Breast cancer is one of the most common cancers among women in South Africa and the second leading cause of cancer death after lung cancer. Women have a 12% chance of developing invasive breast cancer and a 3% chance of dying from it [52]. Breast cancer treatment entails surgical removal of the tumor that is followed by chemotherapy with or without radiation [53]. However, the notorious effect of such treatments has in many cases exacerbated the overall diminishing health and quality of life of the recipients. The greatest challenge of cancer research is to advance the efficacy of cancer diagnosis, monitoring, and treatment. The focus has been turned to the preparation and application of nanoparticles for cancer therapy with emphasis on the dose-enhancement effect of AuNPs and the therapeutic potential of AuNPs in radiation therapy of cancer [34,54].

Dose enhancements can be accomplished by introducing a high-atomic-mass (Z) contrast agent, such as gold, that provides the greatest probability for photo-interactions by the photoelectric effect when radiated by low-energy X-rays [32,33]. The photoelectric interactions produce photoelectrons and Auger electrons, which introduce a localized dose enhancement in cells. The Auger effect is greatest in atoms of medium and high atomic mass, wherein the Auger electrons act as α-particles, producing high local ionization density damage. In conjunction with increasing knowledge on the properties and effects of AuNPs, they are being investigated as potential tools for cancer therapy. In this study, cellular uptake of AuNPs and their effect on cell viability was investigated. In order to demonstrate a possible interaction between the X-rays and AuNPs, an exceptionally high concentration of AuNPs was used, and the chromosomal damage and changes in cellular kinetics were studied (Figure 7).

Surface plasmon resonance (SPR) is determined from absorption and scattering spectroscopy, and is found to depend on the shape, size, and dielectric constants of both the metal and the surrounding material [20]. Thus, increased particle size is noticeable, with a peak shifting to a longer wavelength, whilst increased width of absorption spectra corresponds to the size distribution range [55].

The absorbance profile of 5 nm and 10 nm AuNPs, as measured using UV-vis spectrophotometry, is shown in Table 1. The λmax was between 500–565 nm, with SPR at 525 nm for both types of AuNPs. The UV-vis absorption peaks observed corresponds to the excitation of SPR in AuNPs and provides as an affirmation of their presence. This gives a brilliant red color to the AuNPs, which varies in relation to their size.

Z-potential provides essential information on the dispersion of nanoparticles, as the charge is an indication of the repulsion forces between particles that can be utilized to predict long-term stability of the nanoparticles in suspension. Z-potential, DLS, and PDI determination were used to assess the charge and hydrodynamic size, as well as the size distribution width, of the citrate-coated AuNPs (Table 1). The negative Z-potential charge of the AuNPs (from −24.5 mV for 5 nm AuNPs to −23.2 mV for 10 nm AuNPs) represents the necessary repulsive forces for the particles to remain stable in solution (Table 1). PDI data were employed to determine the size width distribution of the AuNPs. The PDI measurements ranged from 0.202–0.329, which suggests that the AuNPs were uniform in size (Table 1). The Z-average size/DLS (diameter in nm) was 38.12 nm and 48.50 nm, respectively, for 5 nm and 10 nm AuNPs (Table 1). It is known that smaller AuNPs tend to aggregate [56,57], and, true to form, the hydrodynamic size of the AuNPs was greater than the core size measured by TEM. AuNPs were coated with citrate ions, which act as stabilizing agents via electrostatic repulsion [58], but could still aggregate in a solution with sufficiently high ionic strength or low pH [59,60].

Previous studies reported that DNA DSBs induce H2AX phosphorylation, and the number of γH2AX foci is directly related to the number of radiation-induced DNA DSBs [61]. The γH2AX foci formation can be considered as a consistent and quantitative marker of radiation-induced DNA DSBs [62]. H2AX is one of several genes coding for histone H2A, which can undergo phosphorylation, acetylation, and ubiquitination to regulate the cellular events [63]. γ-H2AX phosphorylation assay is a quantification technique by definite immunofluorescent staining that has been widely used to visualize the individual amount of DNA DSBs, and is described as a highly sensitive method to monitor DSB induction and kinetics repair [64]. The isolated lymphocytes incubated with AuNPs were radiated with low doses (1 Gy X-ray and 1 Gy p(66)/Be neutron) to maintain accuracy in automated foci. At doses above 1 Gy, the 1:1 correlation between γ-H2AX foci and DNA DSBs becomes unreliable due to the clustering of DSBs into repair domains, complicating accurate quantification. This approach ensures that the measured foci remain representative of individual DNA damage events rather than merged repair clusters [64]. The number of foci per cell increased slightly compared to the control. A significant increase in the number of foci was noticeable for cells treated with AuNPs followed by X-ray and p(66)/Be neutron radiation, respectively, compared to the control (Figure 3).

A study conducted by Wiwanitkit et al. (2009) demonstrated that 9 nm citrate-coated AuNPs could enter lymphocytes. Since lymphocytes have no phagocytosis activity [65], the only mechanism is the direct penetration of AuNPs into the cytoplasm of the lymphocyte [66], substantiating the usefulness of nanoparticles as novel drug delivery systems to lymphocytes [67]. According to this study, intracellular AuNPs could be observed in about 90.4 ± 8.5% of lymphocytes with no morphology changes when compared to control lymphocytes. However, the AuNPs could be seen in the cytoplasm, but not in the nucleus. In our study, the AuNPs could be seen mainly in the cytoplasm of MCF-7 and MCF-10A cells (Figure 1 and Figure 2). Wiwanitkit et al. (2009) suggested further research into the penetration mechanism of AuNPs into the cytoplasm of isolated lymphocytes, as the lymphocyte membrane pore size is 4 nm × 2.5 nm, which was much smaller than the 9 nm AuNPs employed in the study [66].

Cell viability assays evaluate the overall toxicity of treatments such as AuNPs on cultured cells, by establishing cell survival and proliferation [68]. It is important to know the dose of treatment required for a specific treatment. Many studies have reported non-toxicity of AuNPs [69], but other researchers found AuNPs to have a toxic effect on cells [70,71]. It is known that metallic Au is non-toxic, but gold chloride or potassium gold cyanide is toxic to organs [72]. Hence, AuNPs are considered to be non-toxic as their cores are inert [73]. Previous studies suggest that cytotoxicity associated with AuNPs is dependent on concentration, side chains, the stabilizer used, surface modifications, the type of toxicity assay, cell line, and physical/chemical properties [74,75,76,77,78]. The variation in toxicity with respect to different cell lines has been observed in human lung and liver cancer cell lines [79]. AuNPs have many side-effects due to the interaction with cell membranes, mitochondria, or the nucleus [78].

Numerous drugs/medications are beneficial at low doses and toxic to cells at high doses. Several studies reported that the cytotoxicity of AuNPs is dose-dependent [80,81,82]. In this study, AuNPs were found to have similar toxicity effects at low doses on the non-malignant cells (CHO-K1 and MCF-10A) when compared to the malignant cell line (MCF-7). At a high concentration of AuNPs (50 μg/mL), a significant reduction (*p* < 0.05) in cell viability was seen in all four cell lines.

Pivodová et al. (2015) conducted a cytotoxicity study of negatively charged AuNPs (−23.4 mV) by a cell viability MTT assay [78]. It was shown that AuNPs do not have a significant cytotoxic effect on normal human dermal fibroblasts (NHDF) and normal human epidermal keratinocytes (NHEK). Previous studies reported that spherical citrate capped AuNPs (21 nm) do not have a toxic effect on human breast cancer cell lines (MCF-7) or human prostate cancer cell lines (PC-3), as well as the spherical citrate capped AuNPs (10–50 nm) are not toxic to human leukemic cells (K562) [76,83].

The latter was noted in cells treated with 50 μg/mL of 10 nm AuNPs. The observation led to the belief that the AuNPs were taken up via receptor-mediated endocytosis. Possible autophagosomes are also observed in MCF-10A (Figure 1). It is uncertain if the goal of these autophagosomes was cell survival or ultimately cell death, as it is known that a link between autophagy and apoptosis exists [84]. The cell viability showed AuNPs can adversely affect cellular proliferation, probably by interacting with essential cell components including the nuclear membrane of the cell, mitochondria, or nucleus. Adverse effects include organelle or DNA damage, oxidative stress, apoptosis mutagenesis, and protein up/down-regulation [74,85,86].

Propidium iodide (PI) staining detected by flow cytometry was utilized to investigate the effects of 5 and 10 nm AuNPs followed by attenuated 6 MV 2 Gy X-ray radiation. The latter allowed for the quantification of DNA content. Table 2 shows the cell progressions of the abovementioned cells, expressed as a percentage (%).

A buildup was observed at the G2M phase in the cell cycle of 5 nm AuNP-irradiated CHO-K1 and MCF-7 cells (Table 2). No increases were noted in MCF-10A cells (Table 2). DNA content analysis showed a significant increase in the number of 10 nm AuNP-treated CHO-K1 cells in the S phase (42.4%) and in the G2/M phase (48.1%) after radiation (Table 2). Exposure to 5 and 10 nm AuNPs, respectively, increased the number of cells in the G2/M phase (45.7% and 47.1%) of the MCF-7 cell cycle, when compared to control cells in the G2/M phase (33.7%). However, a significant increase (63.2%) was observed in the G2/M phase of the MCF-7 cells treated with 5 nm AuNPs and radiated (Table 2). According to Roa et al. (2009), AuNPs accumulated in prostate cancer cells (DU-145 cells) at the G2/M phase via the activation of both checkpoint kinases (CHK1 and CHK2) [87]. Thus, these results suggested that AuNPs may be utilized to enhance the radiotherapeutic sensitization effect in cancer therapy. p53, cyclin E, cyclin A, and cyclin B were identified as being the major mediators of AuNP-induced cell cycle changes, resulting in a significantly increased expression of cyclin E and cyclin B1, and decreased expression of cyclin A.

Cyclin E is a G1 cyclin and is the foremost regulator of the G1/S transition, wherein Cyclin E binds to CDK 2, leading to the formation of cyclin E-CDK 2 complex, which progresses the cell from the G1 to the S phase, described as the G1/S transition [88,89,90]. As cells become dedicated to initiating division, the cells commence DNA replication and proceed to the S phase. These cells rely on specific checkpoints and can delay mitotic entry. DNA synthesis is mediated by the ATM and ATR protein kinases and CHK1 and/or CHK2, in which CHK1 is activated at the replication fork arrest in the S phase, whilst CHK2 is activated by damaged DNA detected during interphase [91]. The CHK1 is necessary to avoid DNA damage with regards to replication stress during the S phase, whilst CHK2 is vital for the detection and repairing of DNA damage during interphase periods [92]. The checkpoint kinases caused the arrest of cell cycle progression via the regulation of cyclin-CDK activation [90].

As a result, AuNPs (5 and 10 nm) in this study could have inhibited the expression of cyclin E to accelerate the G0/G1 phase and consecutively caused the accumulation of cells in the G2/M phase. After Roa et al. (2009) treated the DU-145 cells with glucose capped AuNPs (Glu-AuNPs), the expression of cyclin B1 by the cells was significantly increased (*p* < 0.05). This increase in cyclin B1 formed a cell accumulation in the G2/M phase [87]. The buildup in the G2/M phase was noted by the induction of 5 and 10 nm AuNPs that led to DNA damage. DNA damage activates the ”guardian of the genome”, namely p53, which inhibits cyclin B expression and causes cell cycle arrest in the G2/M phase. A therapeutic agent, such as AuNPs, can be utilized to cause an accumulation in the G2/M phase to enhance radiation sensitivity [87,93].

AuNPs are of interest for in vitro and in vivo applications in radiotherapy due to their well-known biocompatibility [94] and effectiveness as radiosensitizers at low energies for the activation of the high K-edge of gold (80 keV) that can lead to the emission of short-range photoelectrons upon irradiation (200–500 kV range) [34,95]. Hainfeld et al. (2004) were the first to show that intravenously administered 1.9 nm untargeted AuNPs accumulated and enhanced the radiation-induced death of mammary carcinomas in mice models when combined with kilovolt (kV) photon radiation [15]. A Monte Carlo study predicted that the theoretical dose enhancement achieved by gold radiosensitization is up to 200% or more [8,96].

As expected, AuNPs reduced the cellular kinetics of all the cell lines (Figure 5). The mean MN frequency for the non-radiated control samples were negligible for all three cell lines. In the event of a radiosensitization effect, the number of MN of attenuated 6 MV X-ray radiated cells treated with AuNPs should be higher than that of the controls (Figure 6). Results showed that the non-malignant CHO-K1 and MCF-10A cells, as well as the malignant MCF-7 cells incubated with AuNPs, were more sensitive to radiation damage. However, the CHO-K1 and MCF-7 cells displayed significantly different interaction indices between the control cells and the 50 μg/mL AuNPs treated and radiated (attenuated 6 MV 2 Gy X-ray) (Figure 2) cells. Therefore, only CHO-K1 and MCF-7 cell lines were used for further experiments in this study.

Apoptosis is characterized by loss of cell-to-cell contact, detachment, cell shrinkage (loss of K^+^ and water) nuclear condensation, inter-nucleosomal DNA cleavage (CAD-activation), nuclear fragmentation, membrane blebbing, and cell-self-fragmentation into apoptotic bodies [97,98]. In contrast to apoptosis (a non-physiological cell death), necrosis can lead to cytoplasm mitochondria swelling, resulting in ATP depletion due to mitochondrial dysfunction [99,100]. Mitochondria swelling was noted in MCF-10A cells incubated with 50 μg/mL of 10 nm AuNPs in TEM micrographs (Figure 1), which could be indicative of the start of necrosis in these cells. The concentration of AuNPs used in this study was exceptionally high, as determination of the radiation interaction indices with gold was the main aim. However, the high concentrations did have a detrimental effect on cell morphology, as mentioned above, and cell viability, especially in the 24 h exposure periods, as shown in the MTT studies.

CHO-K1 and MCF-7 cells were incubated for 4 h with a much lower concentration of AuNPs (2.5 μg/mL), as employed in a study by Jain et al. (2011) to establish if a similar radiation interaction between AuNPs and the X-rays could be obtained [101]. No significant interaction indices were present. This finding differs from the results obtained using the high concentration of AuNPs (50 μg/mL), where a significant interaction between AuNPs and X-rays was obtained, especially in the CHO-K1 and MCF-7 cells.

Ionizing radiation (IR) interacts with DNA either directly or indirectly, which damages cells either directly or indirectly through the production of free radicals, causing DNA single- or double-stranded breaks (DSBs). High-LET radiation damages the DNA directly by breaking hydrogen bonds connecting base pairs, whereas low-LET damages the DNA indirectly through radicals and reactive molecules [102]. As a cell consists of 80% water, IR often generates free radicals, as previously mentioned, by splitting a water molecule (H_2_O) into hydrogen ions (H+), hydroxyl radicals (OH-), or hydrogen peroxide (H_2_O_2_), which could initiate harmful chemical reactions in cells. High levels of ROS can cause damage to macromolecules, such as lipids, nucleic acids, and proteins, leading to the induction lipid peroxidation [103].

A high number of MN within BNCs of CHO-K1 and MCF-7 for both treatments of 50 μg/mL with 1 Gy or 2 Gy p(66)/Be neutron radiation (energy mean of 29 MeV) was observed, respectively. No significant interaction was observed between the AuNPs and neutron radiation. Both CHO-K1 and MCF-7 cell lines, after being treated with AuNPs (5 and 10 nm) followed by 2 Gy p(66)/Be neutron radiation, displayed many MN (Figure 8). In this study, Auger electrons were definitely produced, as well as free radicals and charged species (ROS). Low-LET X-ray-induced Auger electrons were possibly produced in cell lines treated with 50 μg/mL of AuNPs, but a significant interaction between X-rays and AuNPs was only seen in CHO-K1 and MCF-7 cell lines. Auger electrons, which are weakly bound electrons cast out as a result of electronic shell rearrangements, can produce high local ionization density. Several Auger electrons are generally emitted from the same atom simultaneously, causing highly concentrated localized damage [8,104]. However, they travel much shorter distances, usually ~10 nm. The Auger effect is greater in atoms of medium and high Z, such as gold [8].

Different mechanisms of interaction between X-rays and nanoparticles, and neutrons and nanoparticles, are expected according to the chemical nature of the nanoparticles, in this case, AuNPs. Gold (Au) has a high atomic number (Z = 79) that enhances the photoelectric effect, and thus the subsequent emissions of secondary electrons to increase conventional radiation therapy efficacy when bombarded with low-voltage X-rays [105]. The attenuation coefficient (cm^−1^) for 125 kV X-rays for gold is 35.95 and only 6.23 for neutrons [106]. X-ray photons interact with the orbital electrons of atoms of the absorbing matter, namely AuNPs, and give off fast electrons. In contrast, neutrons interact with the nuclei of atoms of the absorbing matter (AuNPs) and set fast recoil protons, α-particles, and heavier nuclear fragments in motion [35,107]. Thus, the lack of interaction between the AuNPs and the neutrons was expected and served as a negative control in this study.

## 4. Materials and Methods

### 4.1. Reagents and Cells

All chemicals and solutions, including dimethyl sulfoxide (DMSO), tetrazolium bromide (3-(4,5-dimethylazol-2-yl)-2,5-diphenyl-tetrazolium bromide, MTT), glutaraldehyde, osmium tetroxide, propylene, resin, uranyl acetate, propidium iodine (PI), and Acridine Orange (AO) were of analytical grade and purchased from Sigma-Aldrich (Johannesburg, South Africa).

Gamma-irradiated fetal bovine serum (FBS) was supplied via GIBCO (Thermo Fisher Scientific, Johannesburg, South Africa). Phosphate-buffered saline (PBS) without Ca^2+^, Mg^2+^ and phenol red were purchased from WhiteSci (Cape Town, South Africa), and Trypsin 10X was purchased from Sigma-Aldrich (Johannesburg, South Africa). Dulbecco’s Modified Eagle Medium (DMEM), Ham’s F-12 Medium and RPMI 1640 Medium for tissue culture, and penicillin-streptomycin were obtained from Thermo Fisher Scientific (Johannesburg, South Africa). Epidermal growth factor (EGF), hydrocortisone, and insulin were purchased from Sigma-Aldrich (Johannesburg, South Africa). The Chinese hamster ovary cells (CHO-K1) were a gift from Prof. J. P. Slabbert, NRF-iThemba LABS (Somerset West, Cape Town, South Africa), whereas human breast adenocarcinoma (MCF-7) and non-tumorgenic human breast epithelia cells (MCF-10A) were purchased from the American Type Culture Collection (ATCC), Maryland, United States of America.

### 4.2. Gold Nanoparticles (AuNPs)

The 5 nm and 10 nm AuNPs were purchased from Sigma-Aldrich (Johannesburg, South Africa). The AuNPs were filtered and stored in sterile 50 mL tubes to ensure that no contamination could occur.

### 4.3. Sample Collection and Isolation of Lymphocytes for γ-H2AX Foci Assay

Peripheral blood samples were collected in sodium heparin blood collection tubes (Cat# 368884 and BD Vactainer^®^ PLUS) from 3 healthy adult volunteers. Donors were non-smokers and had no history of radiotherapy treatment within the last ten years. Ethics approval for the collection of the blood was granted by the Biomedical Research Ethics Committee (BMREC) of the University of the Western Cape (reference number: 15/4/100; original approval date: 19 August 2015), and all experiments and methods were performed in line with relevant guidelines and regulations. In total, CD3^+^ T cells were isolated from peripheral blood using the RosetteSep™ Human T Cell Enrichment Cocktail (Stemcell Technologies) (Vancouver, BC, Canada) by negative selection after density gradient centrifugation (Density: 1.081 g/mL, RosetteSep™ Density Medium, Stemcell Technologies, Vancouver, BC, Canada). Unwanted cells were targeted for removal with Tetrameric Antibody Complexes recognizing CD16, CD19, CD36, CD56, CD66b, and glycophorin A on red blood cells (RBCs), resulting in a highly enriched population of CD3 T-lymphocytes (purity > 98%).

### 4.4. General Cell Culture Procedures

The CHO-K1 cells were cultured using RPMI 1640 medium supplemented with 10% FBS. MCF-7 cells were cultured using Dulbecco’s Modified Eagle’s medium F-12 (DMEM F-12) supplemented with 10% FBS, penicillin (100 μg/L), and streptomycin (100 μg/mL). MCF-10A cells were all cultured using Dulbecco’s Modified Eagle’s medium F-12 (DMEM F-12) and Ham’s F-12 (50:50), supplemented with 5% Fetal Bovine Serum (FBS). Furthermore, the MCF-10A medium was supplemented with EGF (20 ng/mL final concentration), hydrocortisone (0.5 mg/mL final concentration), insulin (10 μg/mL final concentration), penicillin (100 μg/L), and streptomycin (100 μg/mL) [108]. The aforementioned cell lines were cultured under standard conditions at 37 °C, 5% CO_2_ air and a relative humidified atmosphere. Tissue culture flasks, serological pipettes, and filters were obtained from a South American supplier via BIOCOM/Biotech (Cape Town, South Africa).

### 4.5. Characterization of AuNPs

#### 4.5.1. UV–Visible (Vis) Absorption Spectrophotometry

The absorption of both AuNP solutions was measured via UV-vis spectrophotometry for the analysis of SPR to substantiate the presence of AuNPs, in addition to the estimated size and quantity. UV-vis spectra measurements were recorded as a function of wavelength using an Agilent 8453 spectrophotometer (GenTech Scientific Inc., San Diego, CA, USA), with a software program known as UV–visible ChemStation (version G1115A).

#### 4.5.2. Zeta (Z) Potential, Dynamic Light Scattering (DLS), and Polydispersity Index (PDI)

The Z-potential is the main indicator of attractive or repulsive forces between nanoparticles. Therefore, the Z-potential parameter can be used to forecast the stability of the nanoparticles’ dispersion over a long-term period. The DLS measurement represents the hydrodynamic core size of nanoparticles in suspension. In addition, PDI measurements were used to determine the size distribution width of the AuNPs. Z-potential and PDI measurements of AuNPs were determined using Malvern Instruments’ Zetasizer Nano ZS (Malvern, UK). The data were obtained in the phase analysis light scattering mode at 25 °C.

#### 4.5.3. Transmission Electron Microscopy (TEM)

A TEM analysis was done to observe the uptake, as well as the location of AuNPs within the malignant (MCF-7) and non-malignant (MCF-10A) breast cells. Each flask (*n* = 1) of cells was treated with 50 μg/mL of 10 nm AuNP solution for 4 h. Afterwards, the media was removed, and the cells were washed with PBS. To each of these flasks, 2.5% glutaraldehyde diluted in PBS (pH 7.4) was added for fixation of the cells. The cells were harvested through scraping and centrifuged at 1000 rpm for 3 min to obtain a stable pellet.

Post-fixation, cells were washed thrice in 200 mM phosphate buffer for 5 min and stained with 1% osmium tetroxide diluted in 100 mM phosphate for 60 min. Afterwards, the cells were counter-stained with 1% uranyl acetate diluted in 100 mM phosphate. Both treated cell samples were washed with distilled water to remove phosphate ions, and thereafter dehydrated in ethanol (EtOH) (50, 70, 90, and 100%) for 5 min. Ethanol was then replaced with propylene oxide, and the cells were washed in this solution to remove plastic residues. The solution was removed and replaced with 50% propylene oxide and 50% resin solution and dried for 2 h. The 50% solution was removed and replaced with pure resin and dried for an extra 2 h. A 1 μm thick section of each sample was made using a microtome, and deposited on a Formvar-coated 200–300 mesh copper grid and analyzed (TEM JEOL JEM-1011, Cape Town, South Africa).

### 4.6. Experimental Set-Up for Radiation Procedures

All cell samples were irradiated as monolayers in 25 cm^2^ culture flasks or 9 cm^2^ petri dishes, depending on the assay. The irradiation experiments were performed using a special attenuated set-up for the clinical linear accelerator (LINAC) operating at 6 MV peak photon energy mode and with a p(66)/Be neutron beam with a mean neutron energy of 29 MeV at iThemba LABS (Somerset West, Cape Town, South Africa).

#### 4.6.1. X-Ray Radiation

**Figure 9:**
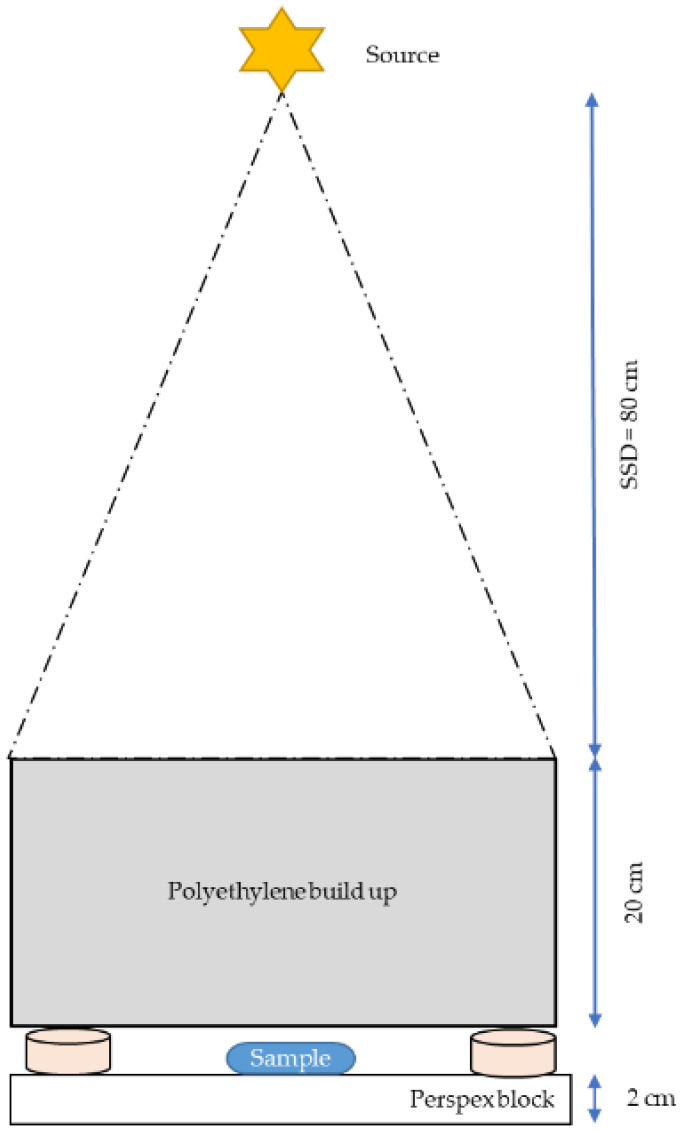
Attenuated 6 MV X-ray experimental set-up.

Samples were irradiated with 6 MV LINAC using a vertical beam (Philips SL 75-5 LINAC) directed downward through a 20 × 20 × 20 cm build-up of material, consisting of 20 cm polyethylene on a 2 cm thick backscatter block of Perspex with the same dimensions (Figure 9). This resembles the beam “softening” technique described by Berbeco et al. and was accomplished by a phantom, mentioned above [34]. All the samples were placed at the machine’s isocenter and orthogonal to the central axis of the beam to ensure that each sample was irradiated with the same dose. The dose rate was 0.6 Gy/min for a 20 × 20 cm^2^ field size at a source-to-surface distance (SSD) of 80 cm for the build-up plate. Following a 4 h incubation period with AuNPs, cells were irradiated with a dose of 2 Gy. Sham-irradiated control samples were included for each assay.

#### 4.6.2. p(66)/Be Neutron Radiation

**Figure 10:**
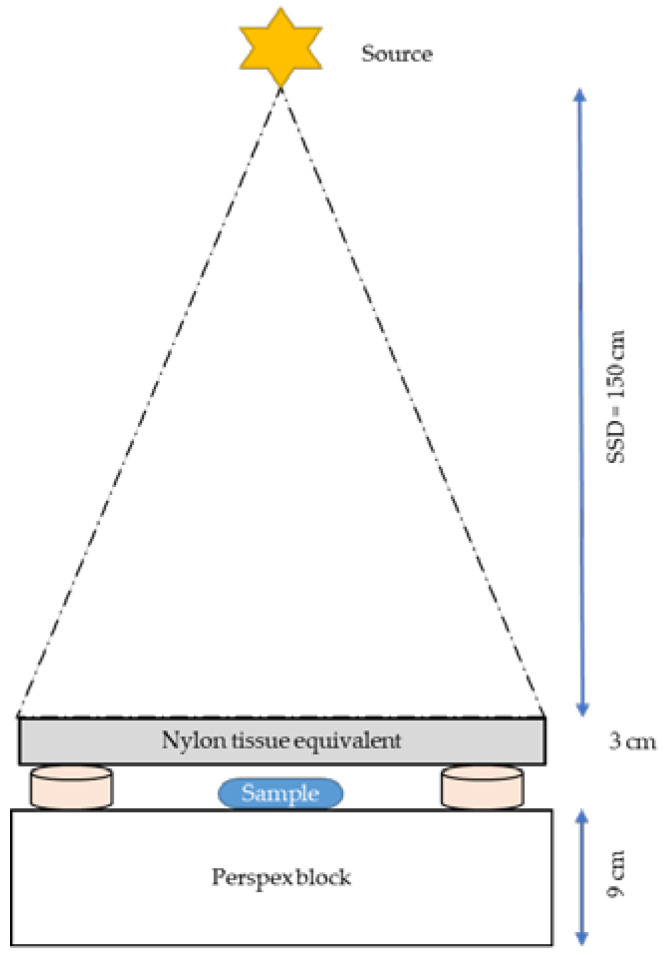
p(66)/Be neutron radiation set-up.

Samples were irradiated with a Scanditronix clinical isocentric gantry, where the neutrons are produced by bombarding a thick Beryllium (Be) target with 66 MeV protons generated by the separated sector cyclotron (SSC) at the iThemba LABS Facility (iTL, Cape Town, South Africa). The beam quality was inferred from the neutron energy spectrum with a fluence-weighted average energy of approximately 29.8 MeV for the 29 × 29 cm^2^ field used [109,110], with build-up material that consisted of one block of nylon (3 cm) [111] on three backscatter blocks of 3 cm thick Perspex (total thickness = 9 cm) (Figure 10). The source-to-phantom surface distance was 150 cm, and the dose rate was 0.5 Gy/min for p(66)/Be neutron radiation. The output factor (1.097 Gy/MU) was measured at the same position as the samples using an Exradin T2 thimble ionization chamber, with a wall made from A-150 tissue-equivalent plastic with a 0.53 cm^3^ active chamber volume flushed with a propane-based tissue-equivalent gas. The ^60^Co calibration factor used for the cross-calibration of the T2 chamber is traceable to the National Metrology Institute of South Africa (NMISA), while calibrations were performed according to the neutron dosimetry protocol as described in the ICRU Report 45 [112].

**Figure 11:**
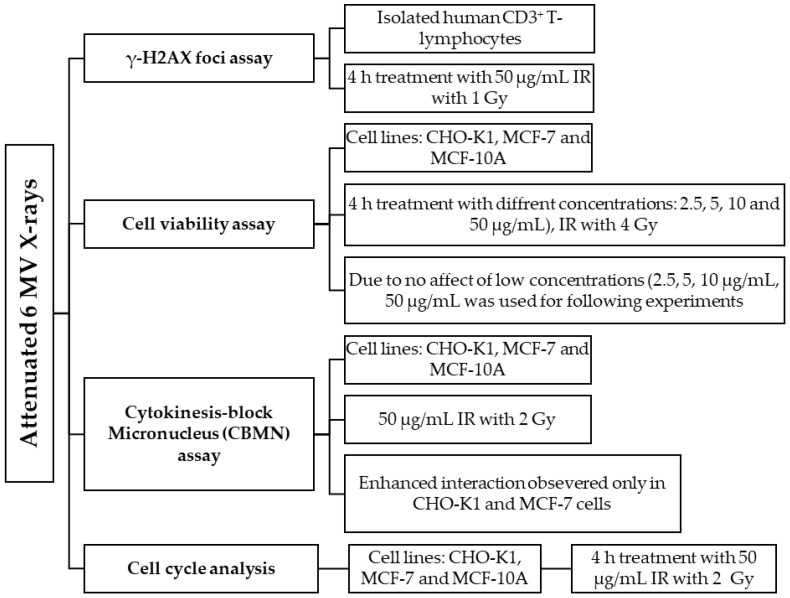
Overview of assays conducted with attenuated 6 MV X-ray radiation. Cells were exposed to a defined dose of attenuated 6 MV X-ray radiation to assess cellular responses.

**Figure 12:**
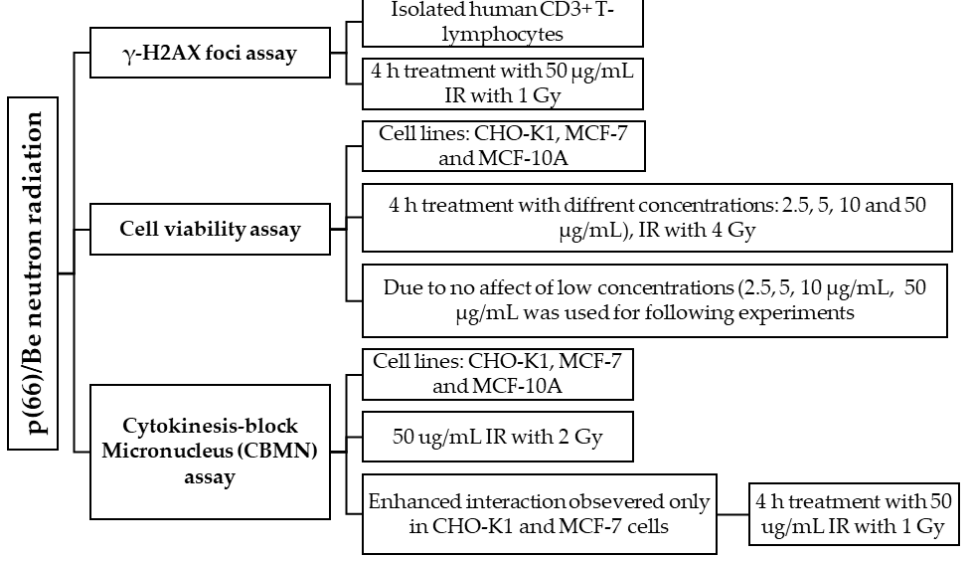
Overview of assays conducted with attenuated p(66)/Be neutron radiation. Cells were exposed to a defined dose of attenuated p(66)/Be neutron radiation to assess cellular responses.

### 4.7. γ-H2AX Foci Assay

γ-H2AX foci assay is a commonly used for the quantitative evaluation of radiation-induced DNA double-strand break (DSB) formation in lymphocytes [113]. The γ-H2AX focus assay was carried out in isolated human CD3^+^ T-lymphocytes to establish radiation damage following a 4 h treatment with 50 μg/mL AuNPs. This assay was performed on isolated CD3^+^ lymphocytes, since these cells are quiescent when they are not stimulated, which results in cleaner images with less background noise in the sham-irradiated control samples and fewer variations between irradiated samples. After incubation with AuNPs of two different diameters (5 nm and 10 nm) and irradiation with 1 Gy X-rays or 1 Gy p(66)/Be neutrons, isolated lymphocyte cells were centrifuged onto poly-L-lysine-coated slides (VWR International, Philadelphia, PA, USA) at a concentration of 6 × 10^5^ cells/mL. The slides were fixed in PBS containing 3% paraformaldehyde (PFA) (Life Technologies, Carlsbad, CA, USA) for 20 min and stored overnight in PBS (4 °C) containing 0.5% PFA. The next day, slides were washed with PBS for 10 min and treated with 0.2% Triton X-100 solution (Life Technologies) in PBS for 10 min. Thereafter, cells were blocked by washing them three times for 10 min in PBS containing 1% Bovine Serum Albumin (BSA) obtained from Sigma-Aldrich (Johannesburg, South Africa). Immunohistochemistry staining was performed using a monoclonal primary antibody (Ab) against γ-H2AX (1:500, Mouse mAb, Life Technologies). Slides were incubated with the primary Ab for 1 h at room temperature. After washing the cells three times in PBS containing 1% BSA, the slides were incubated for 1 h at room temperature with Tetramethyl Rhodamine Isothiocyanate (TRITC) rabbit-anti-mouse antibody (1:1000, Life Technologies) as a secondary antibody. Afterwards, the slides were rinsed three times in PBS and mounted in a solution of Fluoromount purchased from Sigma-Aldrich (Johannesburg, South Africa) containing 2% 4′,6-diamidino-2-Phenylindole (DAPI) (Life Technologies).

Slides were stored in a cool, dark place before image capturing to allow the mounting medium to dry and to avoid fading of the fluorescent signal. Slides were scanned by a Metafer 4 System (Metasystems, Heidelberg, Germany) at iThemba LABS. Images were obtained automatically by using MetaCyte software (version 14.8.1). In each experiment, at least 1000 cells were scored over two slides in randomly selected fields of view.

### 4.8. Cell Viability Assay

Exponentially growing CHO-K1, MCF-7, and MCF-10A cells were plated into flat-bottomed 96-well tissue culture plates and allowed to attach overnight. Thereafter, cells were treated with different concentrations of the two types of AuNPs (2.5, 5, 10, and 50 μg/mL) for different times (4 h non-irradiated, 4 h irradiated with 4 Gy attenuated 6 MV X-rays, and 24 h non-irradiated) in triplicate. The cells were irradiated with 4 Gy X-rays to obtain the maximum effect on cellular proliferation after 4 h. The average of all the experiments is shown as the cell-viability percentage in comparison with the control, which is the untreated cells and considered as 100%. At the end of each treatment period, the toxicity level was measured by adding MTT (5 mg/mL), diluted in PBS (pH 7.4), to each well. The plates were covered with foil, due to light sensitivity, and incubated at 37 °C, 5% CO_2_ air and a relative humidified atmosphere for 4 h. Following incubation, the MTT solution was removed, and the formazan solvent, DMSO, was added. The plate was covered again with foil and placed on a rocker for 15 min. The MTT assay was used to determine the AuNP and radiation effect on the cell viability by measurement of enzymatic reduction of yellow tetrazolium to a purple formazan by cellular mitochondria and detected using PhotoRead Software (version 1) via Apollo LB 913 plate reader (Berthold technologies, Bad Wildbad, Germany) UV-vis spectrophotometer at 570 nm.

### 4.9. Cytokinesis-Block Micronucleus (CBMN) Assay

The CBMN assay is a cytogenic technique in which the aberrations result from cellular damage, both chromosome loss and chromosome breakage, which also provide information on cytotoxicity. The micronuclei (MN) observed in binucleated cells (BNCs) are minute extracellular bodies, separated from the main nucleus, that consist of acentric fragments. Exponentially growing CHO-K1, MCF-7, and MCF-10A cells were seeded on cover slips at 4 x 10^3^ per 9 cm^2^ petri dish and allowed to attach overnight. All the above-mentioned cells were treated with 50 μg/mL with both types of AuNP and subsequently irradiated with attenuated X-rays or p(66)/Be neutron radiation, after which the CBMN assay was performed. Sham-irradiated 0 Gy controls with and without AuNPs were included in all experiments.

Enhanced interaction indices between the AuNPs and attenuated 6 MV 2 Gy X-rays were only observed in the CHO-K1 and MCF-7 cells. Therefore, only these two cell lines were used in the extended study to validate and further investigate the interaction between the AuNPs and radiation.

To determine the interaction indexes of samples the following equation was used, where Unity = 1:

$$Unity=\frac{Combination\;treatment\;of\;micronuclei\;(MN)}{Micronuclei\;\left(MN\right)\;of\;separate\;treatment\;of\;AuNPs+Irradiated\;samples}$$

Further studies included a change in the radiation dose from 2 to 4 Gy X-rays and a decrease in the AuNP concentration from 50 μg/mL. In the same set of experiments, the two cell lines were also treated with AuNPs and irradiated with 1 Gy and 2 Gy neutrons (separate experiments) to establish whether the interaction between AuNPs and attenuated 6 MV 2 Gy X-rays would remain or disappear. After radiation treatment, media with AuNPs was removed, and fresh culture media was added to each cell line. A quantity of 2.25 μg/mL cytochalasin B was added to each experimental sample, to inhibit cytoplasmic division to enable the observation of MN after anaphase division, and incubated for 24 h. Thereafter, the media was removed and the cells were washed with PBS (pH 7.4). This was followed by the addition of 1 mL cold methanol/acetic acid (3:1) for a 5 min fixation. The fixative was removed, and the cells were allowed to air-dry for 15 min. A 0.1% aqueous solution of acridine orange (AO) was prepared for staining, in which a stock solution of 0.24 mM of the stain was diluted in Gurr buffer (pH 6.8) (Gibco Cat# 10582-013 and 1 Gurr buffer tablet supplied from ThermoFisher Scientific was dissolved in 1 L of dH_2_O). The fixed cells on the cover slip were stained with AO for 1 min and rinsed for 1 min in Gurr buffer. The stained cover slips were placed on the centre of the labeled microscope slides, with the excess buffer being blotted, and the slides were sealed with Fixogum rubber cement.

Following previously defined guidelines [114,115,116], scoring of 500 BNCs per slide was done manually using an inverted fluorescence microscope (Zeiss, Oberkochen, Germany), and the FITC filter. Briefly, MN are counted within BNCs to ensure that any possible damage enhancement that has occurred will be observed as MN within the BNCs. The scoring of MN within the BNCs was completed by counting the number of MN within the BNCs and binning them as 0, 1, 2, 3, 4, etc., to generate a ratio of total MN within the entire population of BNCs per slide.

### 4.10. Cell Cycle Analysis via Propidium Iodine (PI) Staining

Each cell line was seeded in a 25 cm^2^ flask at 5 × 10^4^ cells and was allowed to attach overnight. The following day, it was exposed to 50 μg/mL of both 5 nm and 10 nm AuNPs followed by attenuated 6 MV 2 Gy X-ray radiation. Cells were harvested and then centrifuged (Jouan B4 Centrifuge) for 6 min at 900 rpm for pellet formation and washed with PBS (pH 7.4). Permeabilization of the cell membrane was achieved by fixing the cells in 3 mL of ice-cold 99.5% EtOH. The fixed cell suspensions were stored at −20 °C overnight. Subsequently, the EtOH was removed by centrifugation at 1400 rpm for 5 min, and the cells were washed with PBS (pH 7.4) twice. The supernatant was removed without disturbing the pellet, and the sediment was resuspended in 1 mL of the hypotonic DNA staining buffer (20 μg/mL) and stored at 4 °C protected from the light for 30 min, prior to utilization of the BD ACCURI-C6 flow cytometer. For each cell sample, 10,000 events were collected and aggregated cells were gated out.

### 4.11. All Statistical Analyses

Figure 11 and Figure 12 provide an overview of the assays conducted using attenuated 6 MV X-ray radiation and attenuated p(66)/Be neutron radiation to evaluate cellular responses, highlighting the experimental approach and methodologies used to assess radiation-induced effects. Automated scoring of ~1000 lymphocytes was completed via a Metafer 4 System. Statistical analysis was completed via MedCalc program (Version 14.8.1). Multiple comparison graphs were used to visualize and quantify the influence of the AuNP types and radiation on each sample in an experimental group. The Kruskal–Wallis test was performed to identify sample means that are significantly different from each other, and results were considered statistically significant if *p* < 0.05.

Manual scoring of 500 BNCs per slide was completed as described above. The mean cumulative frequency for each experimental group (Control, X-ray, and p(66)/Be neutron radiation) for both types of AuNPs was then calculated, and represented in bar graphs.

For the determination of cellular kinetics, 400 BNCs were counted per condition and expressed as a percentage (%). The percentage of cellular kinetics for each experimental group (Control, X-ray, and p(66)/Be neutron radiation) for both types of AuNPs was then calculated, and represented in bar graphs.

The results from the individual experiments were averaged, and the corresponding standard error of the mean (SEM) calculated. Statistical analysis was performed using Microsoft Office Excel 2019 (Microsoft Corporation, Washington, DC, USA) and GraphPad Prism Software Version 5.01 for Windows (GraphPad Software, San Diego, CA, USA). Multiple comparison graphs with bars were used to visualize and quantify the influence of the AuNP types and radiation on each sample in an experimental group. Kruskal–Wallis tests were performed for all independent comparisons, and results were considered statistically significant if *p* < 0.05. Flow cytometry results obtained was determined by using BD ACCURI-C6 software, version 264.21. All statistical tests were 2-sided, and *p* < 0.05 (*) were considered statistically significant, *p* < 0.01 (**) highly significant, and *p* < 0.001 (***) extremely significant.

## 5. Conclusions

The study investigated the interaction of 5 and 10 nm AuNPs with attenuated 6 MV X-ray and p(66)/Be neutron radiation to enhance radiotherapy. Experiments were conducted in CHO-K1, MCF-7, and MCF-10A cell lines. X-rays, a low LET radiation type, were scattered using build-up material to obtain lower-energy X-rays for AuNP interaction. At 50 μg/mL AuNPs and 2 Gy attenuated X-rays, only CHO-K1 and MCF-7 showed interaction above Unity (U = 1), while lower concentrations (2.5 μg/mL) and neutron irradiation (1 and 2 Gy) did not induce interaction. However, micronucleus (MN) frequencies were higher with combined AuNP-radiation treatment than with either alone, though not consistently. Despite the experimental setup maximizing Auger electron production, expected DNA damage was not observed, likely due to AuNPs being sequestered in lysosomal and autophagosome bodies rather than reaching the nucleus. Cellular kinetics decreased in all experimental conditions, with 50 μg/mL AuNPs reducing proliferation. Flow cytometry showed AuNP-induced G2/M phase accumulation, leading to DNA damage. This suggests that AuNPs may act as radiosensitizers by promoting G2/M arrest, a phase where cells are most sensitive to radiation [117,118].

In conclusion, internalized citrate-coated spherical AuNPs should be quantified via inductively coupled plasma atomic emission spectroscopy (ICP-AES) to explain different results found in CBMN assay and should be further investigated for their radiosensitivity effect before being used in a clinical environment for radiotherapy. In addition, AuNPs can be investigated for how they affect the cell cycle kinetics, using bromodeoyuridine (BrdU) proliferation and apoptosis assays.

## Figures and Tables

**Table 1:** This table shows the characterization of the 5 nm and 10 nm AuNPs.

Characterization of AuNPs	5 nm AuNPs	10 AuNPs
UV-visible (vis) absorption peak	525 nm	525 nm
Zeta (Z) potential	−24.5 mV	−23.2 mV
Hydrodynamic size	38.12 nm	48.50 nm
Polydispersity index (PDI)	0.20	0.33
Size measured by transmission electron microscopy (TEM)	0.005 µm	0.01 µm

**Table 2:** The percentage distribution of all cell lines in different cell cycle phases for controls (untreated), AuNPs, and irradiated AuNP-treated cells in the different cell cycle phases.

Cell Lines	Time Point	Cell Cycle Phase	Treatments					
			Control	Irradiated (IR) Control	50 µg/mL of 5 nm AuNPs	50 µg/mL of IR 5 nm AuNPs	50 µg/mL of 10 nm AuNPs	50 µg/mL of IR 10 nm AuNPs
**CHO-K1**	**4 h**	**G1**	**44.9**	**25.0**	**44.9**	**29.4**	**31.5**	**15.8**
		**S**	**27.4**	**38.2**	**18.2**	**22.3**	**27.3**	**42.4**
		**G2**	**28.2**	**37.9**	**37.3**	**48.1**	**42.5**	**41.9**
**MCF-10A**	**4 h**	**G1**	**68.4**	**57.0**	**64.2**	**52.8**	**60.6**	**59.1**
		**S**	**10.4**	**20.4**	**12.4**	**22.5**	**17.6**	**16.5**
		**G2**	**20.6**	**23.7**	**22.4**	**25.2**	**22.8**	**23.9**
**MCF-7**	**4 h**	**G1**	**47.7**	**26.0**	**42.9**	**23.1**	**29.5**	**27.0**
		**S**	**20.1**	**23.7**	**13.0**	**13.3**	**22.3**	**30.3**
		**G2**	**33.7**	**51.7**	**45.7**	**63.2**	**47.1**	**45.5**

## Data Availability

Data are contained within the article.
